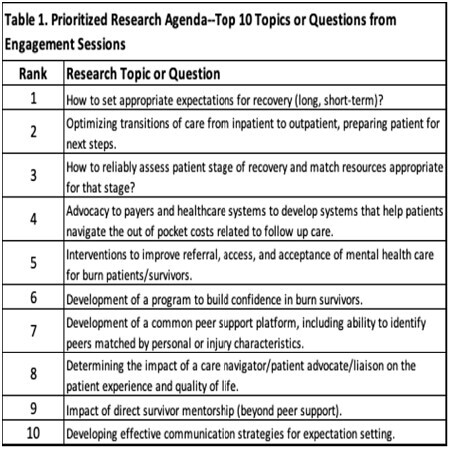# 603 A Burn Survivor- & Burn Community Stakeholder-Generated & Prioritized Research Agenda

**DOI:** 10.1093/jbcr/irae036.237

**Published:** 2024-04-17

**Authors:** Callie M Thompson, Crystal D Webb, Giavonni M Lewis, Ann Marie B Prazak, Kristen C Quinn

**Affiliations:** University of Utah Health, Salt Lake City, UT; University of Utah Health, West Haven, UT; University of Utah, SLC, UT; University of Utah Health, Salt Lake City, UT; University of Utah Health, West Haven, UT; University of Utah, SLC, UT; University of Utah Health, Salt Lake City, UT; University of Utah Health, West Haven, UT; University of Utah, SLC, UT; University of Utah Health, Salt Lake City, UT; University of Utah Health, West Haven, UT; University of Utah, SLC, UT; University of Utah Health, Salt Lake City, UT; University of Utah Health, West Haven, UT; University of Utah, SLC, UT

## Abstract

**Introduction:**

Burn survivors are involved in burn research but typically as a research subject. We believe that the outcomes & impact of burn research can be improved by engaging survivors as collaborators in the planning, implementation, & dissemination of burn research. The goal of the work presented here was to produce the first research agenda generated & prioritized by burn survivors & other invested members of our burn community.

**Methods:**

A research collaborative with a focus of co-production with burn survivors was formed in early 2022. This collaborative is run by governance committees, the majority of whom are burn survivors. Other members of the governance are family members & caregivers of burn survivors, & members of the burn community. The first goal of the collaborative was to form a stakeholder-generated & prioritized agenda for future burn research. The collaborative decided on 4 broad topics for Engagement Sessions (ES) in addition to a 5th topic of “Barriers to Participate in Research”. We then held 16 separate ES with burn survivors & their caregivers. The collaborative leadership then identified specific research questions/topics from the ES transcripts & prioritized those questions via anonymous survey. Notably, all participants in the ES & all committee members were compensated for their time.

**Results:**

In over 26 hours of ES, 117 participants shared their thoughts on 5 broad topics: patient & family education, aftercare, navigating the healthcare system, recovery-physical & psychosocial, & barriers to research participation. From these sessions, 37 research question/topics were identified & ranked. In addition, 19 research barriers were identified & ranked. The top 10 research topics/questions are shown in Table 1. The top 3 barriers to research were: timing for researchers & survivors doesn’t line up (ex: researchers want to study 2 weeks after injury but patients are not in a place to participate), lack of understanding of the expected outcome or benefit, & lack of resources to have bandwidth to participate.

**Conclusions:**

Burn survivors & their caregivers are experts in their lived experiences. By involving them in burn research as collaborators & contributors from the very first steps of research & throughout the continuum of planning, implementing, & dissemination of findings, we believe that the research will be both more successful & more impactful. We have taken the first steps to form the first co-produced collaborative & the first stakeholder generated research agenda for the burn community.

**Applicability of Research to Practice:**

Our research collaborative is a well-informed & invested group that can participate in future studies. This research agenda will be readily available & all are encouraged to answer the questions that our burn survivors have identified to be the most pressing.